# Decreasing trends in tuberculosis cure indicators in Brazil

**DOI:** 10.36416/1806-3756/e20240121

**Published:** 2024-05-08

**Authors:** Marcelo Fouad Rabahi, Marcus Barreto Conte

**Affiliations:** 1. Faculdade de Medicina, Universidade Federal de Goiás - UFG - Goiânia (GO) Brasil.; 2. Faculdade de Medicina de Petrópolis, Centro Universitário Arthur Sá Earp Neto - UNIFASE - Petrópolis (RJ) Brasil.

The aims of tuberculosis therapy are to cure the patient and to reduce the transmission of its etiologic agent, *Mycobacterium tuberculosis*, to both immunosuppressed and immunocompetent people. Although the expected rate of cure of antituberculosis treatment based on laboratory results is remarkably high, this efficacy does not always translate to effectiveness. The effectiveness of tuberculosis treatment corresponds to the successful outcome (cure) under operational conditions. Unsuccessful tuberculosis outcomes (death, loss to follow-up, or treatment failure) are associated with different variables, such as patient-related factors (age, comorbidities, individual vulnerability to toxicities, immune status, nutritional status, alcohol intake, adherence to treatment, drug tolerance, and genetic characteristics), organism/disease presentation-related factors (virulence of the organism, radiological extent of the disease, and susceptibility of the strain), care-related factors (motivational capacity of the staff, access of patients to the health care system, and monitoring and supervision of patients regarding treatment), and treatment-related factors (amount of each drug administered, plasma concentrations of administered drugs, relationship between administered drugs and proteins, clearance, metabolism, and absorption; drug bioavailability of the presentations, drug interactions with other drugs, treatment regimen, bactericidal and sterilizing potency, and drug synergy or antagonism).^1^

A study analyzing the temporal trend of tuberculosis cure indicators in Brazil is presented in this issue of the *Jornal Brasileiro de Pneumologia*.^2^ Pavinati et al.^2^ conducted an ecological time-series study using administrative data of reported cases of tuberculosis nationwide from 2001 to 2022. The authors of the study showed a decreasing trend toward cure indicators for new cases of pulmonary tuberculosis, cases of tuberculosis-HIV coinfection, and cases of retreatment, although results were heterogenous among the Brazilian Federative Units. The influence of the COVID-19 pandemic on the outcomes of this analysis is unmistakable. Nevertheless, a discernible trend was noticed across the three groups studied. Although a first increase in the percentages of cure was noted during the first temporal segment of the investigation, this was followed by a reversal of that trend in the later intervals, characterized by a decrease in cure rates. This observation became more marked in the final segment. These findings suggest that independent of the COVID-19 pandemic, other factors were already at play, contributing to the diminishing rates of tuberculosis cure in Brazil.

These findings go in the opposite direction to the global average data.^3^ According to the WHO report 2023, there is an increasing trend toward the cure indicator worldwide among people newly diagnosed with tuberculosis and enrolled on first-line treatment, with a success rate of 88% for those enrolled in 2021.^3^

Data from a very recent epidemiological report of the Ministry of Health of Brazil showed a decrease in the cure rate of sensitive cases of tuberculosis (from 76.5% in 2013 to 73.8% in 2019).^4^ In 2022, the cure rate was 62%, but one should consider a possible impact of the COVID-19 pandemic (2020-2021) on this result. Also, the rate of unknown outcomes (interruption of treatment/patient referral/unavailable data) increased from 17.9% in 2013 to 20.3% in 2019. In 2022, that was 31.2%, but, again, it could still be a consequence of the impact of the COVID-19 pandemic.

Despite interventions of the Brazilian National Ministry of Health aiming at increasing the cure rate and adherence to treatment, such as directly observed treatment, short-course (DOTS) strategy, fixed-dose combination (FDC) tablets, among others, earlier studies cited in the manuscript^2^ have already suggested a decrease in the cure rate over the years.^5^ ^,^ ^6^ Furthermore, an ecological study published in 2017 performed an interrupted time-series analysis of secondary data from the Brazilian Tuberculosis Case Registry Database between January of 2003 and December of 2014 and showed a decrease in cure rates and an increase in treatment abandonment rates.^7^

As all ecological studies, findings of Pavinati et al.^2^ should be interpreted cautiously. However, given the conclusion of different authors who have observed a constant downward trend in the cure rate as well as a constant increase in the rates of unfavorable outcomes in Brazil, which is clearly in the opposite direction to the global average trend of cure rates worldwide according to the WHO, the conclusion of the authors against current public policies should be observed with all due attention by the public authorities responsible for conducting public health policies in Brazil.
